# Evaluating Pull-Out Strength of Barbed Suture In Vitro by Using Porcine Tissue and Polydimethylsiloxane (PDMS)

**DOI:** 10.3390/polym14112170

**Published:** 2022-05-27

**Authors:** Wei Hong, I-Cheng Chen, Chen-Ying Su, Cherng-Kang Perng, Hsu Ma, Hsu-Wei Fang

**Affiliations:** 1 Department of Chemical Engineering and Biotechnology, National Taipei University of Technology, No. 1, Sec. 3, Zhongxiao E. Rd., Taipei 10608, Taiwan; t107739013@ntut.org.tw (W.H.); icchen.ntut@mail.ntut.edu.tw (I.-C.C.); chenying.su@mail.ntut.edu.tw (C.-Y.S.); 2 Accelerator for Happiness and Health Industry, National Taipei University of Technology, No. 1, Sec. 3, Zhongxiao E. Rd., Taipei 10608, Taiwan; 3 Division of Plastic and Reconstructive Surgery, Department of Surgery, Taipei Veterans General Hospital, 19F, No. 201, Sec. 2, Shi-Pei Street, Beitou Dist, Taipei 11217, Taiwan; ckperng@gmail.com; 4 School of Medicine, National Yang Ming Chiao Tung University, No. 155, Sec. 2, Linong St., Beitou District, Taipei City 11221, Taiwan; 5 Department of Surgery, National Defense Medical Center, No.161, Sec. 6, Minquan E. Rd., Neihu Dist., Taipei City 11490, Taiwan; 6 Institute of Biomedical Engineering and Nanomedicine, National Health Research Institutes, No. 35, Keyan Road, Zhunan Town, Miaoli County 35053, Taiwan

**Keywords:** thread lift, barbed suture, pull-out strength, PDMS, facial rejuvenation, holding capacity

## Abstract

Using barbed thread lifting for facial rejuvenation has become popular these days due to its minimally invasive procedures with reduced complications. However, only limited studies regarding its mechanical properties for face suspension were published. The aim of this study was to evaluate suture-holding ability regarding its facelift property, and different specimens were tested in order to establish an in vitro model. Fresh porcine tissue and the synthetic material polydimethylsiloxane (PDMS) were selected to simulate human skin for evaluating barbed suture pull-out strength by the universal material testing machine. The results showed that the pull-out strength of barbs between different porcine tissues varied without consistency. By contrast, PDMS (30:1) showed more consistent pull-out strength in each testing, and the average maximum load force was close to porcine tissue. Furthermore, after submerging barbed sutures in PBS for 0 days (T0), 7 days (T7) and 14 days (T14), a trend of decreased average maximum load force, displacement and force of 1.5 mm/2 mm/3 mm displacement could be detected by in vitro testing with PDMS (30:1). These results provide support for using PDMS (30:1) to evaluate suture pull-out strength and holding/lifting capacities in vitro to obtain consistent and objective information for evaluating substantial equivalence of devices. The established in vitro method could be used for the future development of barbed thread lifting technology.

## 1. Introduction

Facial ageing is an unwanted process, and the features include facial volume loss, appearance of folds and wrinkles, deepening of nasolabial fold, droopy eyelids, etc. There has been a growing trend in aesthetic medicine to counter signs of ageing for several decades, and the procedure can be approximately divided into traditional facelift surgery and non-operative procedure. For example, traditional subcutaneous facelift involves a separation of skins and underlying tissues, tightening and repositioning of the supporting structures and excision of excessive skin. However, the risk of anesthesia, infection, poor wound healing, hematoma, facial nerve injury, temporary or permanent hair loss, are unavoidable [1,2]. Alternatively, fillers, botulinum toxin injections, thread lifts or laser abrasion are relatively less invasive with reduced complications. Among them, thread lift for facial rejuvenation has become more and more popular due to its minimally invasive property, ease of application, least number of risks, immediate results and rapid recovery [3,4,5].

The barbed suture was first used for cosmetic application to lift the drooping facial tissue by Sulamanidze et al. [6]. Since then, numerous kinds of threads developed for minimally invasive aesthetic surgery procedures have been studied for human clinical practice [7,8]. Thread lifting modality involves the passage of sutures placed into the deeper layers of face while the barbs along the sutures act as hooks to support and elevate the superficial musculoaponeurotic system (SMAS), resulting in lifting effects. Inserted barbed threads also induce inflammatory reaction, providing a bio-stimulatory effect for collagen formation, leading to an appearance of tightening. Nevertheless, most studies addressed only the efficacy and safety of the threads [9,10,11,12,13,14,15,16,17], and there have been only limited studies regarding their pull-out tension strength for face suspension. Sasaki et al. represented the first investigation to evaluate and compare holding displacement tension, slippage tension and pull-out tension of various suspension suture systems [18]. However, the limited number of cadavers and the difference between fresh-frozen and living tissues were the major limitations of this method. Living animals could be alternatives for an in vivo suture-holding test; however, to improve animal welfare and perform humane animal research by following the principles of the three Rs (Replacement, Reduction and Refinement), living animal models should also be avoided or reduced. Therefore, to establish an in vitro method to evaluate suture-holding ability regarding its facelift property is required and necessary.

In this study, we utilized both fresh porcine tissue and polydimethylsiloxane (PDMS) for suture suspension and established an in vitro method to evaluate suture pull-out tension. The main goal of this study was to assess consistent and objective information of suture tension by this in vitro method for the future development of barbed suture technology.

## 2. Materials and Methods

### 2.1. Barbed Thread Sample

**Figure 1:**
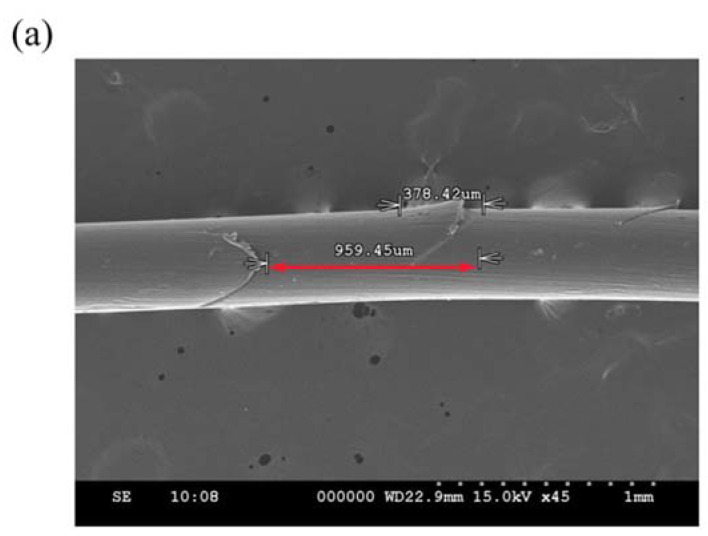

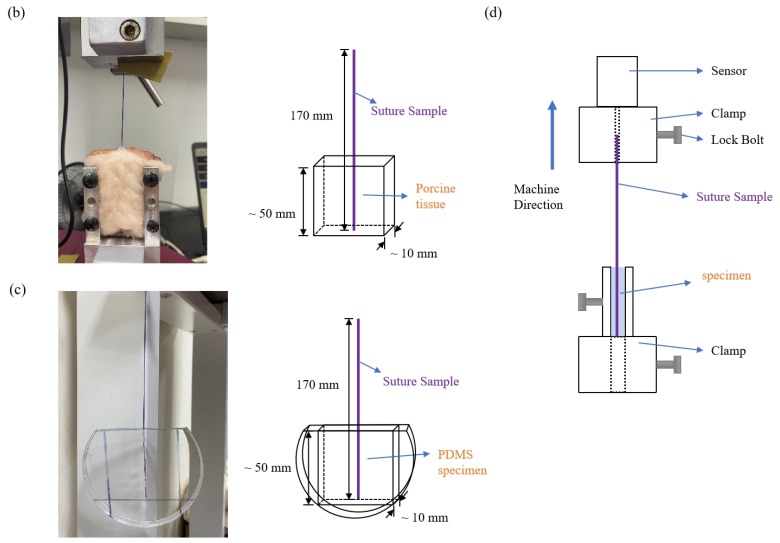
(**a**) The magnified image of the barbed suture. The distance between the barbs is 0.95 mm, on average (red double arrow). (**b**) A photo/diagram of porcine specimen inserted with suture sample. The tissue sample was cut into a rectangular prism (50 mm × 50 mm × 10 mm) for the pull-out test. (**c**) A photo/diagram of PDMS specimen inserted with suture sample. The PDMS specimen was molded and cured in a Petri dish. (**d**) For suture pull-out test, the specimen and suture were set up as the graph shows.

The barbed thread used was Miracle Thread Knotless Tissue-Closure Device (Diamond Biotechnology Co., Ltd.; Taipei, Taiwan) of size USP 1. The device comprised dyed (violet) poly(p-dioxanone) (PDO, (C_4_H_6_O_3_)_X_), and the barbs on the device were arranged bidirectionally, at a 0.95 mm distance, on average (Figure 1a).

### 2.2. Porcine Tissue Specimen Preparation

Fresh porcine tissues from pigs sacrificed in the early morning on testing day were purchased from local traditional market. Tissues from hind leg were selected for the experiments because the structure of the fascia from this part was more homogeneous and flatter, which made it easier for handling. For preparation of the specimen, the fresh porcine tissue was cut into a rectangular prism of approximately 50 mm width, 50 mm length and 10 mm thickness by a scalpel (Figure 1b). Tissue samples were used only on the day of purchase, and specimens were tested in five replicates for each experiment.

### 2.3. Polydimethylsiloxane (PDMS) Specimen Preparation

PDMS specimen was prepared according to the manufacturer’s instructions (SLYGARD 184 Silicone Elastomer; Dow Corning; Midland, MI, USA). Briefly, the base reagent and curing reagent (10:1 or 30:1) were thoroughly mixed for 20 min, then poured into a 90 mm Petri dish as a mold to make a disk with a radius of 45 mm and a thickness of 10 mm (~50 g) (Figure 1c). The PDMS specimen was then cured for 2 h in the oven at 70 °C and was ready to use. Specimens were tested in five replicates for each experiment.

### 2.4. Pull-Out Test of Barbs’ Strength

To set up the pull-out test, the blunt needle (cannula) with the barbed thread was firstly inserted into the fascia of porcine specimen or PDMS specimen to guide the thread, then removed from the tissue or PDMS, leaving the barbed thread inside the specimen. Next, the porcine tissue specimen or the PDMS specimen inserted with barbed thread was fixed in the universal material testing machine (YM-H35, Yang Yi Technology Co., Ltd.; Tainan, Taiwan), as Figure 1d shows. The thread sample was clamped in the upper jaw, while the specimen was clamped in the lower jaw of the testing machine, and the distance between the two clamps was 100 mm. The upper clamp was driven at a speed of 100 mm/min, and all the data (e.g., force, displacement) were recorded using QCTech3_A2 software.

### 2.5. In Vitro Degradation Profile of Barbed Suture Sample

The thread samples were kept in a serum bottle containing phosphate-buffered saline (PBS), then placed in a cell culture incubator at 37 °C. After leaving for 0 days (T0), 7 days (T7) and 14 days (T14), the thread samples were taken out for further analyses [19,20]. Threads were tested in five replicates for each treatment.

### 2.6. Tensile Strength of Barbed Thread

The thread was fixed in the universal material testing machine (YM-H35, Yang Yi Technology Co., Ltd.; Tainan, Taiwan), and the gauge between the two clamps was 130 mm. The test machine drove the clamp at a speed of 250 mm/min until the thread broke. All experiments were performed in five replicates.

### 2.7. Scanning Electron Microscopy (SEM)

The thread samples were fixed to the metal support and coated with a thin layer of gold by a sputter coater (Ion Sputter E101, Hitachi, Tokyo, Japan). Representative images of threads from different degradation levels were obtained using S-3000H microscope (Hitachi, Tokyo, Japan) at 15 kV under low vacuum conditions.

### 2.8. Statistical Analysis

All the obtained data were statistically analyzed with two-tailed *t*-test to make an allowance for comparisons. A value of *p* < 0.05 was considered significant.

## 3. Results

### 3.1. Comparison of the Barbed Thread Strength between Different Specimens Using the Pull-Out Test

**Figure 2:**
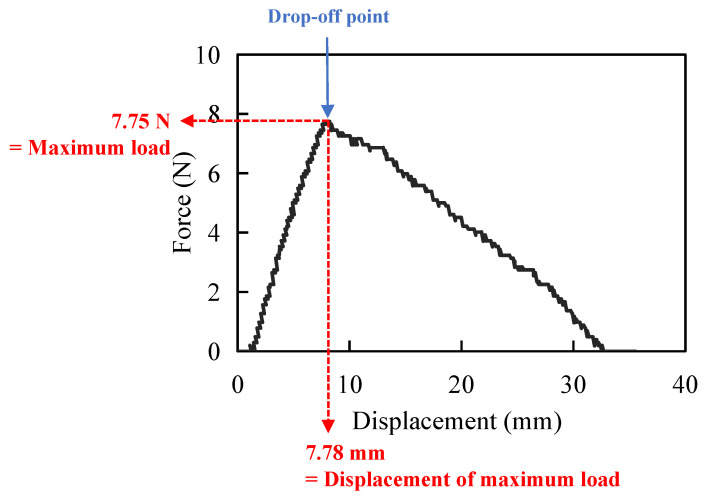
This is an example of a graphic showing the result of the pull-out test. The force is increasing during the pull-out procedure when the barbs hold the specimen. When the barbs fail, the force drops off (blue arrow), and the force of this point is defined as “maximum load”; the moving distance is “displacement of maximum load”.

When the barbed thread was gradually pulled out from the specimen by the universal material testing machine to measure its pull-out strength, the barbs firstly grasped the specimen, which resulted in increasing force during the testing procedure, indicating the increasing holding capacity of the barbs (Figure 2). Then, there was a significant drop-off point (Figure 2, blue arrow), while the barbs initially slipped and released from the fixation site. We defined the force as the “maximum load” and the distance as “displacement of maximum load” (Figure 2, red arrows) at the drop-off point. The force would gradually decline after the significant drop-off point, while the whole suture was pulled out, and the barbs lost their holding capacity.

**Figure 3:**
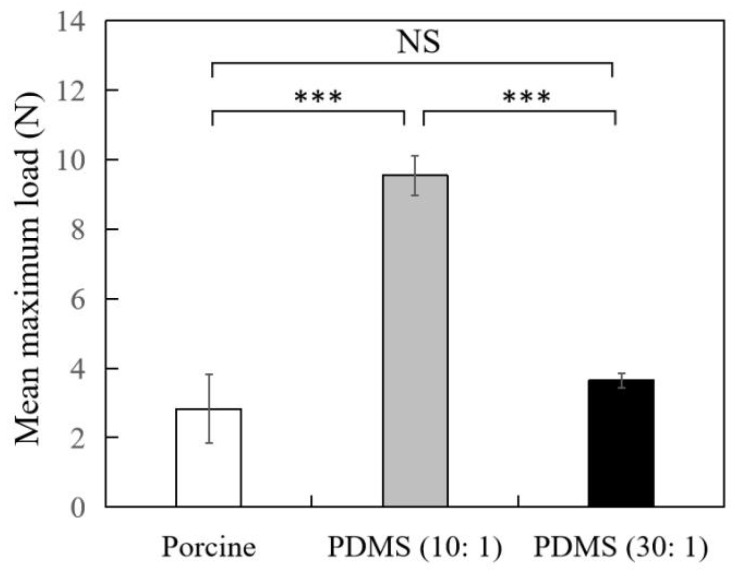
Pull-out test of barbs’ strength with different specimens (porcine, white bar; PDMS 10:1, gray bar; PDMS 30:1, black bar). *** *p* < 0.001 when compared with PDMS 10:1. NS, not significant.

To search for ideal materials for evaluation of the suture pull-out strength, we firstly used fresh porcine soft tissue as specimens to simulate ‘‘facelift’’ soft tissues. As Figure 3 (white bar) and Table 1 show, the average maximum load value of porcine tissue was 2.82 ± 0.987 newton (N), and the variations between tissue samples were large. To avoid individual variation between animal tissues, we then used synthetic material PDMS as substitutes to compare the results. At first, we prepared PDMS by mixing the base and curing reagent with 10:1 ratio, according to the instruction of manufacturer, and the average maximum load value of PDMS 10:1 was 9.55 ± 0.570 N for the pull-out test. Although the standard deviation of the pull-out force of PDMS 10:1 sample was smaller than porcine tissues (0.570 N vs. 0.987 N), the pull-out results derived from PDMS were more consistent (Figure 3, gray bars; Table 1). The hardness of the PDMS 10:1 specimen was too high, and the average maximum load was significantly higher than porcine tissue (*p* < 0.001), which could not simulate the soft tissue. To adjust the hardness of PDMS, the base and curing reagent ratio was changed to 30:1. The average maximum load of PDMS 30:1 was 3.65 ± 0.214 N (Figure 3, black bars; Table 1), which is close to the porcine group (*p* = 0.14, not significant), and this could be used for further pull-out experiments.

### 3.2. In Vitro Degradation Profile of Barbed Suture Samples Evaluated by Pull-Out Test Using PDMS 30:1

**Figure 4:**
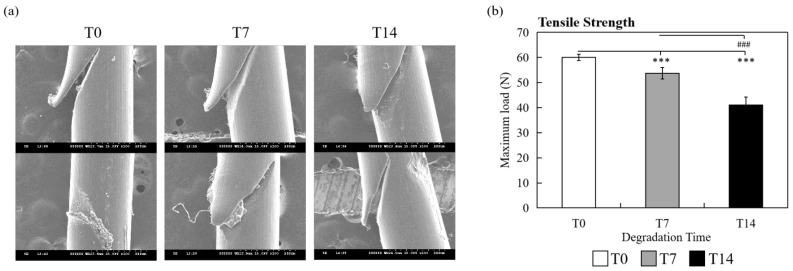
(**a**) The SEM images of the barbed threads after being immersed in PBS for 0 day (T0), 7 days (T7) and 14 days (T14). (**b**) Tensile strength test of T0 (white bar), T7 (gray bar) and T14 (black bar) samples. *** *p* < 0.001 when compared with T0 samples; ^###^ *p* < 0.001 when compared with T7 samples.

To confirm whether PDMS 30:1 is suitable as a specimen for the pull-out test, barbed threads with different degradation days (T0: 0 day, T7: 7 days, T14: 14 days) were examined. Firstly, the changes of threads from T0, T7 and T14 were observed by SEM. In spite of different immersion days, only subtle degradation of thread surface was identified, as shown in Figure 4a. Though the morphology changes were not obvious, the tensile strength test showed that the average maximum load of T0 samples was larger than T7 and T14 samples (T0: 60.01 ± 1.271 N; T7: 53.70 ± 2.257 N; T14: 41.11 ± 3.173; T0 vs. T7, *p* < 0.001; T0 vs. T14, *p* < 0.001; T7 vs. T14, *p* < 0.001), indicating a significant correlation between degradation level of the threads and tensile strength (Figure 4b).

**Figure 5:**
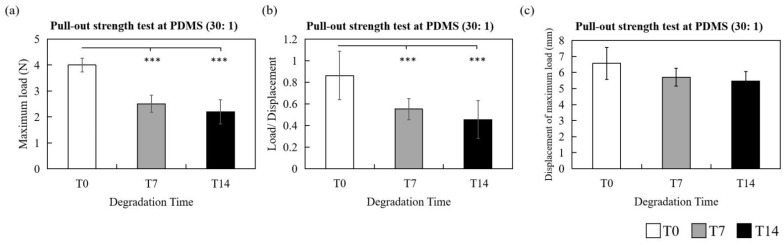
Comparison of (**a**) average maximum load (N), (**b**) load/displacement and (**c**) average displacement of maximum load (mm) of T0 (white bars), T7 (gray bars) and T14 (black bars) samples by pull-out test with PDMS (30:1) specimens. *** *p* < 0.001 when compared with T0 samples.

Next, the pull-out strength experiments developed in this study were performed with PDMS 30:1 as testing specimens to evaluate the holding capacity of T0, T7 and T14 samples. The mean maximum load values (i.e., the maximum holding capacity of barbs) of pull-out tests for T0, T7 and T14 were 4.00 ± 0.323 N, 2.51 ± 0.323 N and 2.20 ± 0.469 N, respectively (Figure 5a). The average maximum holding capacity of T0 barbs was significantly much higher than T7 and T14 (T0 vs. T7, *p* < 0.001; T0 vs. T14, *p* < 0.001). The slope values (load/displacement) indicating the holding capacity under the consistent speed of lifting (100 mm/min) before the drop-off point of T0, T7 and T14 samples were also analyzed. The slope of T0 was notably larger than T7 and T14 (load/displacement of T0: 0.86 ± 0.225; T7: 0.55 ± 0.098; T14: 0.46 0.176; T0 vs. T7, *p* < 0.001; T0 vs. T14, *p* < 0.001) (Figure 5b). Moreover, the average displacement of maximum load (i.e., the maximum lifting capacity of barbs) from T0, T7 and T14 samples was compared. As presented in Figure 5c, displacement decreased during the period of immersion time (T0: 6.57 ± 1.001 mm, T7: 5.71 ± 0.556 mm, T14: 5.48 ± 0.594 mm), though not significantly.

**Figure 6:**
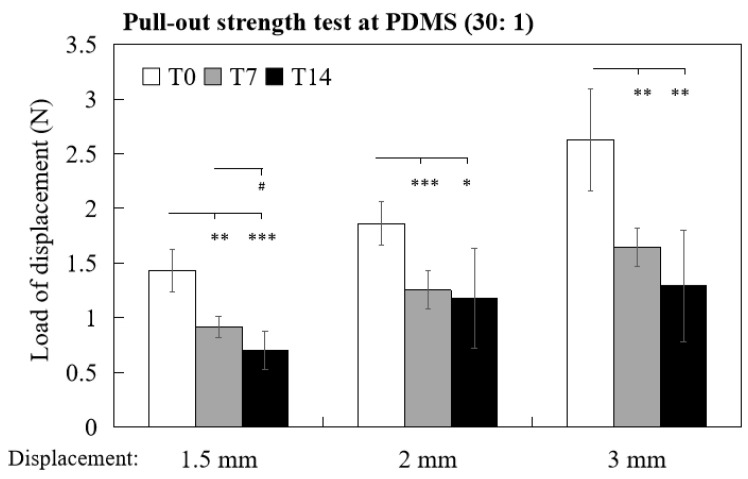
Comparison of average load (N) of 1.5 mm, 2 mm and 3 mm displacement from T0 (white bars), T7 (gray bars) and T14 (black bars) barbed threads by pull-out test with PDMS (30:1) specimen. * *p* < 0.05, ** *p* < 0.01 and *** *p* < 0.001 when compared with T0 samples; ^#^ *p* < 0.05 when compared with T7 samples.

According to Nestor’s clinical study of midface suspension, the surface area of 0.5 mm~1.5 mm lift was analyzed using 3D surface imaging system to evaluate the lifting effect of the device [21]. To mimic the actual facial lift situation, loads of 1.5 mm, 2 mm and 3 mm displacement (i.e., holding capacity of the barbs for lifting 1.5/2/3 mm) were determined by in vitro pull-out test, and a similar trend was noted where the load values of T0 samples of 1.5 mm, 2 mm and 3 mm displacement were all significantly higher than T7 and T14 samples (Figure 6). The loads of 1.5 mm displacement of T0, T7 and T14 samples were 1.43 ± 0.191 N, 0.92 ± 0.098 and 0.71 ±0.175, respectively (T0 vs. T7, *p* < 0.01; T0 vs. T14, *p* < 0.001; T7 vs. T14, *p* < 0.05). The loads of 2 mm displacement of T0, T7 and T14 were 1.86 ± 0.196 N, 1.26 ± 0.175 and 1.18 ± 0.460, respectively (T0 vs. T7, *p* < 0.001; T0 vs. T14, *p* < 0.05). The loads of 3 mm displacement of T0, T7 and T14 were 2.63 ± 0.467 N, 1.65 ± 0.175 and 1.29 ± 0.511, respectively (T0 vs. T7, *p* < 0.01; T0 vs. T14, *p* < 0.01). Altogether, by the pull-out test with PDMS 30:1 as a specimen, the holding and lifting capacities of barbed thread could be evaluated and compared in vitro.

## 4. Discussion

Barbed threads, also called knotless tissue closure devices, were mainly applied to soft tissue closure in laparoscopic surgery [22,23,24,25]. Numerous in vitro studies were dedicated to investigating wound closure ability of barbed sutures, but only few in vitro studies discussed about their facial suspension property [26,27,28]. Most of the published reports evaluated the ability of suture suspension by clinical trials [21,25,29,30]. The results from those clinical studies showed only the effects of rejuvenation; however, the information of barbed sutures regarding mechanical properties was missing in these trials, which may produce unknown risks for patients or unnecessary tests. To address the issue regarding thread lifting ability in vitro, we established a pull-out testing system and examined different materials, including fresh porcine tissue and PDMS, as specimens for optimizing the in vitro method to evaluate the pull-out strength of barbed sutures.

### 4.1. Pull-Out Test Using Animal/Human Tissue as Specimens to Evaluate Barbs’ Strength

The study established by Sasaki et al. [18] was inspiring, and it provided helpful information on suture-holding properties using human cadavers. However, the major limitations were the small number of available human cadavers and the difference between fresh-frozen and living tissues, which made it not feasible for large-scale testing. In addition, the pull-out force was measured manually by pulling the tension meter without fixed speed, bringing in uncertain factors involved and difficulties for comparison of different samples. In another study conducted by Angelos et al., the SMAS tissue was harvested from 12 fresh-frozen cadaver heads and prepared for testing reinforcement with or without poly-4-hydroxybutyric acid (P4HB) absorbable mesh. Though care was taken to minimize the variability, large variations were still observed between the SMAS samples [31]. Therefore, the initial objective of this study was to replace human tissues with fresh animal tissues, which could be obtained easily with desirable amounts, qualities and sizes.

In thread lifting surgery, surgeons pass barbed sutures into deeper layers of the face, and the barbs along the sutures act as hooks to anchor and elevate SMAS [29,32,33,34]. To imitate the procedure, we inserted the suture sample into the fascia in fresh porcine tissue to simulate the facelift in SMAS tissue. Contrary to expectations, with fixed size and section of porcine tissue, the results of the pull-out test were not consistent (Figure 3), and there are several possible explanations for such a broad range of maximum load between tissue samples. For example, different storage conditions, freshness of the pork or different composition of the tissue may have influenced the testing results.

### 4.2. Pull-Out Test Using Synthetic Materials as Specimens to Evaluate Barbs’ Strength

To reduce the confounding factors of porcine tissue, we replaced the animal model with synthetic materials. PDMS is a widely used biomaterial in medical practice because of its ability to simulate human body parts [35,36], low cost and ease of handling. Indeed, consistency results were achieved by utilizing PDMS as specimen for the barb pull-out test (Figure 3), and this could be attributed to the homogeneity of the material. To simulate animal tissue, PDMS 30:1 was more suitable for further testing of the in vitro pull-out method due to its similar pull-out force compared with porcine soft tissue (Figure 3 and Table 1), and there was a significant correlation between the pull-out strength and barbed sutures with different levels of degradation (Figure 5 and Figure 6; T0, T7, T14). Though the SEM image of the barbed sutures immersed in PBS for 7 days and 14 days did not show any obvious damage on the surface, the tensile strength of the threads was decreased after immersion (Figure 4), and the difference of their pull-out strength could be detected with the in vitro method established in this study. Interestingly, in the study of Sasaki et al., the slippage tension of Contour PDO suture system measured with human cadaver head was 16.5 ounces (equivalent to 5 N), and our results showed the average maximum load of PDO T0 sample measured with PDMS was 4 N. The results obtained from PDMS are similar with those mentioned in earlier study conducted with human midfaces, suggesting PDMS (30:1) as a promising material for the in vitro test [18].

The complete resorption of the barbed suture material PDO was supposed to take approximately 6 months (unpublished data). For further exploration, the barb pull-out strength of longer degradation time, samples with various number of barbs or different suture materials would be examined and compared using this model in our future studies. In addition to PDMS, it is also worth exploring novel synthetic biomaterials for the in vitro pull-out test to mimic thread lifting environment. For instance, combining biomaterials with additive manufacturing technique to develop novel testing specimens for simulating the extracellular matrix structure in skin with fiber, collagen, elastin, blood and lymphatic vessels could be our future research direction [35,36,37,38].

### 4.3. Clinical Implications of In Vitro Pull-Out Evaluation

In recent years, many innovative suture systems have been quickly developed, and it is crucial to evaluate the holding and lifting capacities of the threads before conducting facelift surgery. In terms of facelift, the bidirectional barbs serve to anchor and support the thread in fibrous tissue in an elevated position. The maximum load of the pull-out test represents the resistant force and the holding capacity of the barbs, while displacement of the thread during the pull-out procedure corresponds to the face-lifting capacity of the thread. In Dr. Nestor’s study, they used a 3D surface imaging system to measure the amount of facial lift from the patients who received midface suspension treatment by Silhouette InstaLift^TM^ sutures [21]. Statistically significant and meaningful changes were observed when patients had lifts of 1.5 mm or greater (<2 mm). In this study, the forces of 1.5 mm, 2 mm and 3 mm displacement were also analyzed, and the results showed that the holding capacities of 1.5 mm/2 mm/3 mm were T0 > T7 > T14 (Figure 6), providing the support for the usage of the in vitro method established herein. Hence, it is convincing that different parameters of the in vitro pull-out method conducted by PDMS (30:1) shown in Figure 5 and Figure 6 can be used to quantify and compare the holding/lifting capacities of different suture devices. A proper analyzing method offered by this study can be applied for evaluating the substantial equivalence of different suture systems to correlate the results with potential clinical effects. For example, when the displacement of maximum load from different tested threads is larger than the standard (e.g., 1.5 mm, according to Nestor’s clinical study [21]), the devices are considered to reach the criteria and be of substantial equivalence.

## 5. Conclusions

In this study, we developed an in vitro testing method with PDMS (30:1) specimen to determine the pull-out strength and holding/lifting capacities of barbed suture, and this established method offers objective information for pre-clinical evaluation of barbed suture for its facelift purpose.

## Figures and Tables

**Table 1:** The pull-out strength tested by different specimens.

	Maximum Load (N)					Mean
Porcine	2.35	1.96	2.75	2.55	4.51	2.82 ± 0.987
PDMS (10:1)	9.61	8.83	9.32	10.4	9.61	9.55 ± 0.570
PDMS (30:1)	3.63	3.73	3.33	3.92	3.63	3.65 ± 0.214

## Data Availability

The data presented in this study are available on request from the corresponding author.
